# Novel Biomarkers for Alzheimer's Disease: Plasma Neurofilament Light and Cerebrospinal Fluid

**DOI:** 10.1002/alz70856_096481

**Published:** 2025-12-24

**Authors:** Daniel Naawenkangua Abukuri

**Affiliations:** ^1^ University of Ghana, Accra, Greater Accra, Ghana

## Abstract

**Background:**

Neurodegenerative disorders like Alzheimer's disease present growing public health challenges. Clinical diagnosis faces limitations, making biomarkers increasingly vital for research, clinical trials, and patient assessments. Current biomarkers, including amyloid‐β peptide, tau proteins, cerebrospinal fluid levels, and neuroimaging techniques, while effective, are often invasive and costly. Blood‐based biomarkers offer a noninvasive, affordable, and reliable alternative for monitoring neurodegeneration.

**Method:**

This review explored recent advancements in blood‐based biomarkers, focusing on plasma neurofilament light (NfL) and CSF NfL, and their application in diagnosing and monitoring Alzheimer's disease.

**Result:**

The review identified strong associations between Alzheimer's disease (AD) and key biomarkers. Core CSF markers, including T‐tau, *p*‐tau, and Aβ42, showed significant diagnostic value, with *p*‐tau elevated by 181% and Aβ42 reduced by 46% in individuals with mild cognitive impairment (MCI) due to AD. Elevated plasma and CSF NfL levels correlated with a 25–40% reduction in [18F]FDG uptake and a 2.5% annual hippocampal volume loss, compared to 1% in controls. Plasma *p*‐tau, combined with the Aβ42/Aβ40 ratio, demonstrated over 85% diagnostic accuracy for symptomatic AD stages, while CSF NfL predicted cognitive decline with 80% accuracy. Although blood NfL is not AD‐specific, it reliably detects neurodegeneration and monitors treatment efficacy. These findings highlight the promise of integrating blood‐based biomarkers with CSF markers for improved AD diagnosis and management.

**Conclusion:**

Blood‐based biomarkers, particularly NfL and plasma *p*‐tau, represent a transformative advancement in the diagnosis and management of Alzheimer's disease. Their integration with consistent CSF biomarkers (T‐tau, *p*‐tau, Aβ42, and NfL) is strongly recommended for both clinical applications and research, offering a more accessible and reliable approach to addressing the challenges of Alzheimer's disease diagnosis and care.

